# Analysis of Conserved Glutamate and Aspartate Residues in *Drosophila* Rhodopsin 1 and Their Influence on Spectral Tuning*

**DOI:** 10.1074/jbc.M115.677765

**Published:** 2015-07-20

**Authors:** Lijun Zheng, David M. Farrell, Ruth M. Fulton, Eve E. Bagg, Ernesto Salcedo, Meridee Manino, Steven G. Britt

**Affiliations:** From the Departments of ‡ Cell and Developmental Biology,; § Pharmacology, and; ¶ Ophthalmology and Rocky Mountain Lions Eye Institute, University of Colorado, Anschutz Medical Campus, School of Medicine, Aurora, Colorado 80045

**Keywords:** Drosophila, photoreceptor, retina, rhodopsin, site-directed mutagenesis, color perception

## Abstract

The molecular mechanisms that regulate invertebrate visual pigment absorption are poorly understood. Studies of amphioxus G_o_-opsin have demonstrated that Glu-181 functions as the counterion in this pigment. This finding has led to the proposal that Glu-181 may function as the counterion in other invertebrate visual pigments as well. Here we describe a series of mutagenesis experiments to test this hypothesis and to also test whether other conserved acidic amino acids in *Drosophila* Rhodopsin 1 (Rh1) may serve as the counterion of this visual pigment. Of the 5 Glu and Asp residues replaced by Gln or Asn in our experiments, none of the mutant pigments shift the absorption of Rh1 by more than 6 nm. In combination with prior studies, these results suggest that the counterion in *Drosophila* Rh1 may not be located at Glu-181 as in amphioxus, or at Glu-113 as in bovine rhodopsin. Conversely, the extremely low steady state levels of the E194Q mutant pigment (bovine opsin site Glu-181), and the rhabdomere degeneration observed in flies expressing this mutant demonstrate that a negatively charged residueat this position is essential for normal rhodopsin function *in vivo*. This work also raises the possibility that another residue or physiologic anion may compensate for the missing counterion in the E194Q mutant.

## Teaser

**Background:** Rhodopsin absorption is regulated by interactions between the retinal chromophore and amino acids within the opsin apoprotein.

**Results:** Site-directed mutagenesis of conserved residues has only modest effects on *Drosophila* Rhodopsin 1 absorption.

**Conclusion:** The counterion may reside at another site within the protein or other mechanisms may compensate.

**Significance:** Determining the molecular basis for rhodopsin spectral tuning is essential for understanding rhodopsin function and evolution.

## Introduction

**FIGURE 1.**
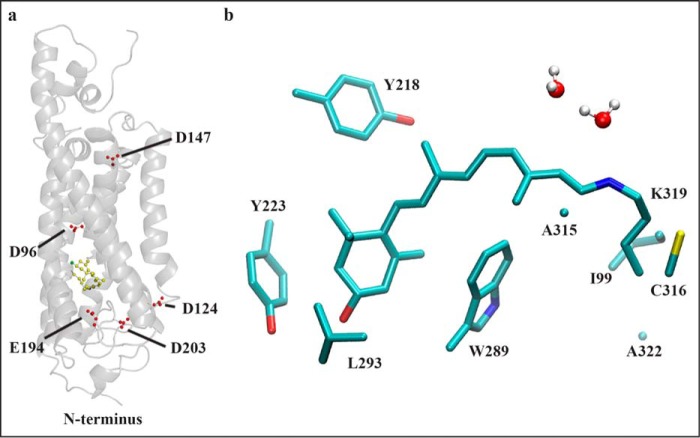
**Molecular modeling of wild-type *Drosophila* Rhodopsin 1.** *a*, model of wild-type *Drosophila* Rh1 pigment as a ribbon diagram showing the residues mutated in the present study (Asp-96, Asp-124, Asp-147, Glu-194, and Asp-203, shown as single letter amino acids). The 3-hydroxy-11-*cis*-retinal chromophore is displayed in *yellow*. The 3-hydroxy oxygen atom of the chromophore is displayed in *green*. The model is based on the squid rhodopsin crystal structure (75). *b,* model of wild-type *Drosophila* Rh1 in the region of the chromophore. Amino acid side chains within 2.5 Å of the lysine-bound Schiff base chromophore are shown. The 3-hydroxy-11-*cis*-retinal chromophore is shown in *light blue* with the Schiff base nitrogen in *dark blue* and the 3-hydroxyl oxygen in *red*. Hydrogen atoms are omitted except on water molecules, which are shown in *white*. Sulfur atoms within amino acid side chains are shown in *yellow*. The amino acid side chains shown: Ile-99, Tyr-218, Tyr-223, Trp-289, Leu-293, Ala-315, Cys-316, Ala-322 correspond to Met-86, Met-207, Phe-212, Trp-265, Ala-269, Ala-292, Phe-293, and Ala-299 within bovine rhodopsin, respectively.

The visual pigment rhodopsin is a unique G-protein-coupled receptor that is activated by the conformational change of a covalently attached chromophore rather than the binding of a diffusible transmitter, drug or hormone (1). In both vertebrates and invertebrates, rhodopsin consists of an 11-*cis-*retinal chromophore that is bound to the opsin apoprotein via a protonated Schiff base (Fig. 1*a*). Upon light absorption the chromophore isomerizes from 11-*cis* to all-*trans*, inducing conformational changes in the opsin that produce activated metarhodopsin (2).

Interactions between the retinal chromophore and amino acid side chains in the opsin protein tune the λ_max_ of the chromophore (Fig. 1*b*) (3). Studies have shown that Glu-113 (bovine position) within the third transmembrane helix serves as the retinylidene Schiff base counterion in vertebrate visual pigments (4–6). Negatively charged Glu-113 serves to stabilize the protonated Schiff base and allow the delocalization of electrons through resonant structures of the conjugated retinal polyene system, thereby shifting the absorption of the UV absorbing chromophore and UV absorbing protein to longer wavelengths (7). Removing the negative charge of the counterion from the binding pocket deprotonates the chromophore and yields a UV absorbing pigment (4–6).

In contrast, the comparable amino acid in the visible light absorbing invertebrate pigments is Tyr, or Phe in the UV absorbing pigments. In previous work, we demonstrated conclusively that this substitution is not responsible for the difference in absorption between invertebrate UV and visible absorbing pigments, and that the tyrosine present in the visible absorbing pigments is unlikely to function as a counterion (8). Studies of squid retinochrome have indicated that a conserved Glu-181 (bovine position) is the counterion for this non-G-protein-coupled receptor retinal photoisomerase (9). Similarly, the same group has also shown that Glu-181 in amphioxus (*Branchiostoma belcheri*) rhodopsin and peropsin also functions as the counterion in these pigments (10).

This has led to the proposal that during the course of evolution the location of the counterion has been displaced from Glu-181 to Glu-113 (10). This concept is supported by two findings. First, the loss of the counterion at Glu-181 in amphioxus rhodopsin can be substituted by introduction of Glu at Tyr-113 (Y113E,E181Q double mutant, mutants are referred to by the single letter amino acid designation of the wild-type residue followed by its position number followed by the single letter amino acid designation of the introduced residue) (10). Second, the counterion of the activated photoproduct of bovine rhodopsin is Glu-181 (11), and the photoproduct counterion of amphioxus rhodopsin is also Glu-181 (10). This demonstrates that the counterion in bovine rhodopsin switches from Glu-113 to Glu-181 following photoactivation, whereas in amphioxus the counterion in both rhodopsin and its activated photoproduct is Glu-181. This indicates that whereas either Glu-113 or Glu-181 can function as the amphioxus rhodopsin counterion in the ground state, only Glu-181 serves as a counterion in the activated photoproducts of both amphioxus and bovine rhodopsin.

**FIGURE 2.**
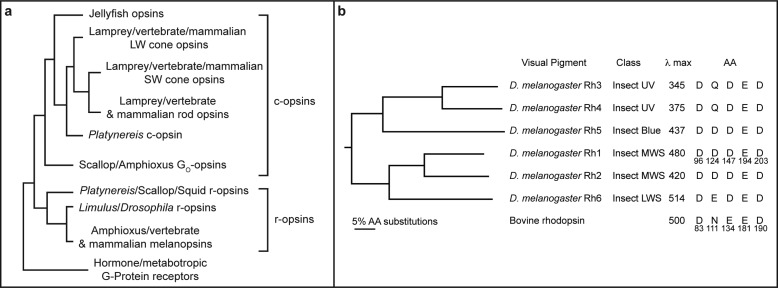
**Phylogenetic relationships between metazoan opsins and those of *Drosophila melanogaster*.** *a,* a simplified phylogeny of major groups of opsins is shown in comparison to hormone and metabotropic G-protein coupled receptors. C-opsins that are found in ciliary photoreceptor cells and R-opsins found in rhabdomeric photoreceptor cells are indicated. These typically couple to transducin/G_i_ or G_q_ G-proteins, respectively. The scallop and amphioxus opsins that interact with G_o_ are also indicated. Modified from Fain *et al.* (19). *b,* phylogenetic relationship between Rhodopsins 1–6 of *D. melanogaster*. The tree was constructed from a ClustalW alignment with bovine rhodopsin as an out group. Both neighbor joining and unweighted pair group methods were used. The trees were bootstrapped 1000 replications and each node was supported 100% by both methods. The class of each pigment is indicated, as referred to in Ref. 43. λ_max_ corresponds to the maximal sensitivity of flies expressing each visual pigment in the R1–6 photoreceptor cells and the λ_max_ of bovine rhodopsin is also indicated (26, 76–79). The amino acid residues present at the sites mutated in the current study are indicated, in comparison to the corresponding residues in bovine rhodopsin.

Molecular dynamics studies of the squid rhodopsin structure have suggested that the residue corresponding to Glu-181 functions as the counterion in squid rhodopsin (12). Despite the findings of this model, it is uncertain whether the counterion mutagenesis results in amphioxus are applicable to other invertebrate species. The amphioxus opsin that has been characterized in mutagenesis experiments is part of a family of G_o_-opsins found in ciliary photoreceptors. These cells hyperpolarize in response to light, as the result of the activation of guanylyl cyclase (13) that increases cGMP and opens K^+^ channels (14, 15). By contrast, the rhabdomeric photoreceptors of *Drosophila* and many other invertebrates depolarize in response to light, as the result of the activation of a heterotrimeric G_q_ protein, which activates phospholipase C and two classes of light-sensitive transient receptor potential channels, TRP and TRPL (16) that admit both Na^+^ and Ca^2+^. In addition to their functional differences, phylogentic evidence suggests that the G_o_-opsins diverged from the r-opsins prior to the cnidarian-bilaterian split, over 580 million years ago (Fig. 2*a*) (17–19). These observations demonstrate that there are substantial functional, structural, and phylogenetic differences between the amphioxus G_o_-opsin and the visual pigments of *Drosophila*.

To test whether Glu-181 or another conserved negatively charged amino acid may serve as the counterion in the *Drosophila* visual pigments, we generated a series of mutations in *Drosophila* rhodopsin 1 (Rh1).^2^ The five mutants (Rh1 D96N, Rh1 D124N, Rh1 D147N, Rh1 E194Q, and Rh1 D203N) correspond to Asp-83, Asn-111, Glu-134, Glu-181, and Asp-190, respectively, in bovine rhodopsin (Fig. 2*b*). We found that each of these mutant Rh1 pigments is functionally expressed *in vivo* in transgenic flies. However, none of the mutants display a dramatic shift in color sensitivity to shorter wavelengths, as would be expected for a mutation in a putative counterion.

## Experimental Procedures

### 

#### 

##### Molecular Biology and Morphology

Flies expressing modified forms of Rh1 were generated in a similar manner to that reported previously (8). Briefly, the gene encoding Rh1 was modified by site-directed mutagenesis using inverse PCR with Pfu DNA polymerase, and DpnI digestion of the methylated template (20, 21). The sequence of the mutagenized fragment was confirmed and the fragment was then subcloned into a 5.4-kb genomic fragment containing the entire Rh1 promoter and coding region. The modified Rh1 was subcloned into the *y*^+^ marked P-element vector “C4” obtained from Pam Geyer (University of Iowa) (22). The construct was injected into *y w; sr ninaE^17^* mutant embryos, and multiple independent P-element-mediated germline transformants were obtained (23). Western blot analysis was performed as described (47). The immunoblot PVDF membrane (Bio-Rad) was incubated simultaneously with mouse monoclonal anti-Rh1 antibody (4C5, Developmental Studies Hybridoma Bank) (24) and rabbit polyclonal anti-actin (Abcam ab1801) overnight at room temperature. The immunoreactive proteins were detected with polyclonal goat anti-mouse IgG (H+L) conjugated to IRDye® 800CW and polyclonal goat anti-rabbit IgG (H+L) conjugated to IRDye® 680CW (both from LI-COR Biosciences). The blots were scanned with an Odyssey Infrared Imaging System (LI-COR Biosciences). Epon-embedded retinal cross-sections were performed as previously described (25).

##### Electrophysiology and Microspectrophotometry

Spectral sensitivity was measured as previously described (8). Electroretinograms and spectral sensitivity recordings were performed on transgenic animals expressing modified forms of rhodopsin in either a *ninaE^17^* background or in a modified *norpA*; *ninaE^17^* mutant background. The latter strain also contained an additional transgene driving the *norpA* cDNA in the R1–6 photoreceptor cells under the control of the Rh1 promoter. This background strain allows the activity of the modified pigment to be examined without interference from the R7 and R8 cells that are not affected by the *ninaE* mutation (26).

Microspectrophotometry (MSP) was performed as previously described (26). A high intensity adapting light was used to photoconvert the visual pigment from its rhodopsin (R) to its metarhodopsin (M) state. The transmission spectrum of each state was measured and a difference spectrum was calculated as previously described (26).

##### Nomogram Curve Fitting

Rhodopsin and metarhodopsin theoretical absorption spectra were calculated from sensitivity and difference spectra as previously described (8, 26), using the spectral shape of the rhodopsin α-band absorption described by the following log normal function,

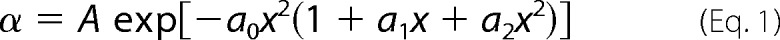
 where

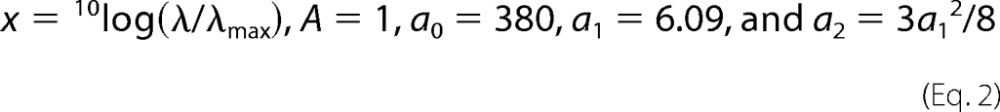


In the case of curve fitting metarhodopsin absorption spectra to the difference spectra measure by MSP, the *R* form absorption was fixed to that determined electrophysiologically (Table 1).

##### Homology Modeling and Dynamics Methods

A protein model for Rh1 was generated using molecular dynamics methods similar to those reported by Ramos *et al.* (27), as we have previously described (28). The structure of wild-type *Drosophila* Rh1 (Swiss-Prot accession P06002) (29) was generated using PHYRE (30, 31) and differs from the original sequence by deletion of residues 1–6 and 242–254. The structure was minimized 5000 steps using NAMD (32) and aligned to squid rhodopsin (PDB entry 2z73) (33) using STRAP (34). The retinal molecule from squid rhodopsin was placed into Rh1 at lysine 319 creating a lysine-bound retinal (LYR-319). The retinal molecule was modified to 3-hydroxy-retinal using VMD (35). Topology and parameter NAMD input for LYR-319 was modified from previous studies (36–41). Internal water molecules were placed using DOWSER (42) followed by a 6000-step minimization. The protein was embedded in a membrane of 1-palmitoyl-2-oleoylphosphatidylchlorine with VMD. Full solvation with TIP3 water molecules, neutralization, and addition of sodium and chloride ions with performed with VMD. The resulting model was 93 Å × 94 Å × 97 Å containing 12,525 water molecules. Full particle mesh Ewald calculations for electrostatics were used for all simulations. To relax the system, a 20-ps run was performed with the protein backbone and LYR-319 fixed. This was followed by a 50-ps run with the protein backbone and LYR-319 residue harmonically constrained. Next, a 50-ps run was performed with LYR-319 residue harmonically constrained, and then finally a 2-ns run was performed with no constraints.

## Results

To test whether Glu-181 or another conserved negatively charged amino acid may serve as the counterion in the *Drosophila* visual pigments we performed an alignment of the six-well characterized *Drosophila* opsins and generated a phylogenetic tree to evaluate their relatedness (Fig. 2*a*). A comprehensive phylogenetic analysis of invertebrate pigments has been reported (43). The *Drosophila* visual pigments represent almost all of the major classes of visual pigments within the invertebrate lineage including ultraviolet, blue, middle wavelength-sensitive, and long wavelength-sensitive pigments (Fig. 2*b*). The phylogenetic relationship between pigments of different spectral types is conserved in most other species (*e.g.* insect UV and blue pigments are derived from the same lineage).

In addition to Glu-181, we identified four other conserved sites that contain negatively charged amino acids in all or most of the *Drosophila* visual pigments. The site corresponding to bovine rhodopsin Asn-111 is the one position that is not conserved in all *Drosophila* pigments and contains Gln in Rh3 and Rh4 and Glu or Asp in the other opsins. The other three sites, corresponding to bovine opsin Asp-83, Glu-134, and Asp-290, contain Asp in all of the *Drosophila* visual pigments. Of these sites, mutagenesis of Asp-83 and Glu-181 have demonstrated significant spectral shifts in vertebrate pigments, reviewed in Ref. 44.

To evaluate the structural relationships of these amino acids within the context of the *Drosophila* Rh1 protein and their relationship with the chromophore and Schiff base-bound lysine residue, we generated a structural model of wild-type Rh1 based on the crystal structure of squid rhodopsin (PDB entry 2z73) (33) using molecular dynamics methods. As expected, the overall topology of the structural model is very similar to that of squid rhodopsin upon which it is based. The root mean square deviation, average distance between the atoms of superimposed proteins, for the pairwise structural model comparisons were as follows: wild-type Rh1 *versus* squid rhodopsin = 3.5 Å, wild-type Rh1 *versus* bovine rhodopsin = 4.2 Å. Overall, this demonstrates a very close fit between the structures as a whole, although the model of wild-type Rh1 shows a higher degree of overlap with the structure of squid rhodopsin upon which it is based, than with the structure of bovine rhodopsin (PDB entry 1U19) (45). The overall topology of the Rh1 protein model is shown in Fig. 1*a* along with the positions of the five charged amino acids described above, Asp-96, Asp-124, Asp-147, Glu-194, and Asp-203. The amino acid side chains within 2.5 Å of the retinal chromophore in the model of wild-type Rh1 are shown in Fig. 1*b*. None of the residues described above are within this distance window. The order of proximity of the closest side chain oxygen of each amino acid to the Schiff base nitrogen in the chromophore: Glu-194 is closest at 6.7 Å; Asp-96 at 13.4 Å; Asp-203 at 14.5 Å; Asp-124 at 15.9 Å; Asp-147 at 29.6 Å.

To investigate the role that these amino acid sites may play as a potential *Drosophila* rhodopsin counterion, we constructed mutant forms of the blue absorbing (Rh1) *Drosophila* opsin, as previously described (8, 28). In these mutants, we replaced the charged residue at the site in Rh1 with an uncharged residue (Asn for Asp and Gln for Glu, (*i.e.* Rh1 D96N, Rh1 D124N, Rh1 D147N, Rh1 E194Q, and Rh1 D203N). We introduced the genes encoding these modified pigments into the germline of *Drosophila* containing the *ninaE* mutation. *ninaE* is a deletion in the endogenous Rh1 gene that is expressed in the R1–6 photoreceptor cells (29, 46). By placing the transgene under control of the Rh1 promoter, we exchange the function of the endogenous wild-type Rh1 pigment with the mutant Rh1 pigment.

**FIGURE 3.**
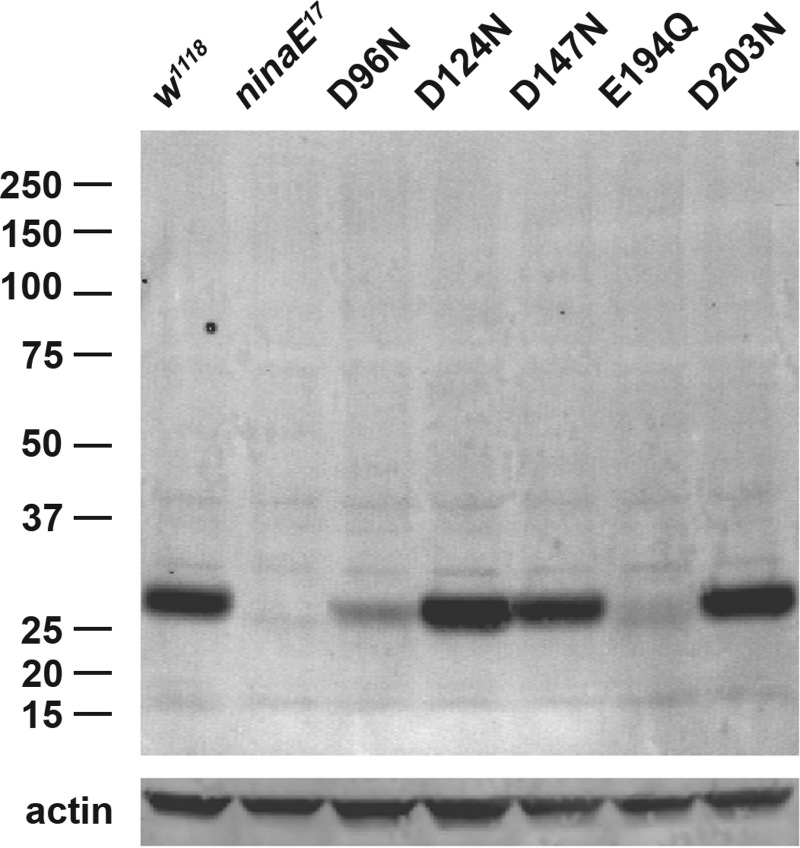
**Steady state expression levels of mutant Rh1 opsins compared with wild-type and the null mutant host strain.** A Western blot of protein extracts from the heads of newly eclosed flies expressing the 5 single amino acid Rh1 mutants is shown. *Drosophila* Rh1 appears as monomer at ∼27 kDa; *numbers* to the *left* of the panel indicate molecular mass markers. Actin was used as a loading control and appears at ∼40 kDa (*lower panel*). Expression levels range from wild-type (D124N, D147N, D203N) to reduced (D96N) or severely reduced (E194Q).

We tested the level of steady state protein expression upon eclosion of flies expressing each mutant pigment compared with the wild-type control (*w^1118^*) and the Rh1 null mutant host strain (*ninaE^17^*) in which each of the transgenes was expressed. As shown in Fig. 3, flies expressing mutants D124N, D147N, and D203N produced normal levels of pigment compared with wild-type flies, whereas the steady state levels found in flies expressing the D96N and E194Q mutants were dramatically reduced. *ninaE^17^* mutant flies show no detectable Rh1 protein, consistent with the 1.6-kb deletion of the gene in this allele (29).

**FIGURE 4.**
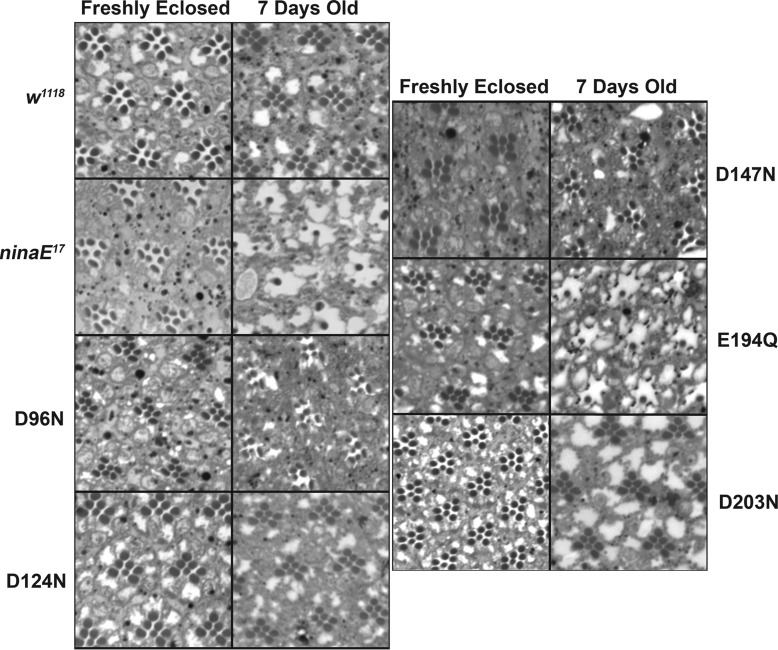
**Retinal morphology of wild-type, *ninaE* null mutant, and Rh1 rhodopsin mutant expressing flies.** Apical cross-sections demonstrate normal retinal morphology upon eclosion for all strains, consisting of normal rhabdomeres of the outer R1–6 photoreceptor cell in each ommatidium surrounding the inner R7 cell rhabdomere. In 7-day-old flies, rhabdomere morphology is maintained in wild-type, *w^1118^*, flies. Rhabdomeres of the R1–6 photoreceptor cells degenerate in *ninaE* mutant flies, because of the loss of the Rh1 opsin in these cells. Rhabdomere morphology at 7 days is completely rescued in flies expressing D124N, D147N, and D203N. At day 7, D96N flies show mild degeneration, whereas E194Q animals show significant degeneration.

To evaluate the functional integrity of the mutant visual pigments, we examined the retinal morphology of animals expressing the mutant pigments and also examined the physiological response of these animals to light by measuring the electroretinogram. As shown in Fig. 4, wild-type flies have well formed rhabdomeres associated with the R1–6 and R7 photoreceptor cells within the distal retina upon eclosion that are maintained at 7 days of age. By contrast, *ninaE^17^* mutant flies, which lack the Rh1 opsin and serve as the host strain for the expression of the mutant pigments in this study, have well formed R1–6 and R7 rhabdomeres upon eclosion, however, the R1–6 rhabdomeres degenerate within 7 days. This is consistent with previous findings that demonstrated a requirement for rhodopsin in rhabdomere morphogenesis in which it organizes the actin cytoskeleton through Rho guanosine triphosphatase (*Drac1*) (48, 49). Ommatidia are seen with a remaining R7 photoreceptor cell rhabdomere, whose expression of Rh3 or Rh4 is unaffected by the *ninaE* mutation. Flies expressing the Rh1 mutants D124N, D147N, and D203N are normal, demonstrating the ability of these mutant pigments to rescue the *ninaE* morphological phenotype. Flies expressing Rh1 mutants D96N and E194Q show mild to severe rhabdomere degeneration at 7 days, respectively.

**FIGURE 5.**
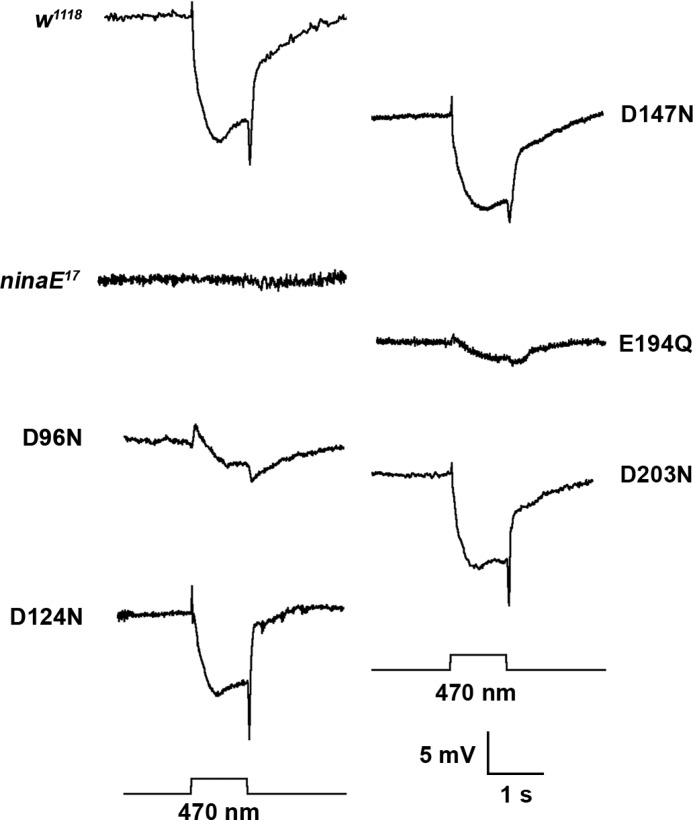
**Rh1 rhodopsin mutants encode functional visual pigments.** Electroretinogram recordings from 7-day-old wild-type controls, *ninaE* mutants, and *ninaE* mutants transformed with transgenes encoding the single amino acid mutant pigments expressed under the control of the Rh1 promoter are shown. *ninaE* mutants do not express rhodopsin in the R1–6 photoreceptor cells. These mutants lack on and off transients and lack a light induced depolarization. Transgenic animals expressing the mutant opsins show proper responses following stimulation at 470 nm, although the amplitude of the response for D96N and E294Q is reduced compared with wild-type. *ninaE* animals were stimulated with 1000-fold higher light intensity. E294Q animals were stimulated with 100-fold higher light intensity. These recordings were performed in a *norpA; ninaE* mutant background in which the *norpA* cDNA was expressed in the R1–6 photoreceptor cells under the control of the *Rh1* promoter. This background strain allows the activity of the modified pigment to be examined without interference from the R7 and R8 cells that are not affected by the *ninaE* mutation (see “Experimental Procedures”).

The light-evoked physiological response of the wild-type, *ninaE^17^* mutant strain and the flies expressing the Rh1 mutant opsins as measured by the electroretinogram at 7 days of age is show in Fig. 5. The response of the wild-type *w^1118^* strain shows a prominent depolarization that is maintained for the duration of the 1-s 470 nm flash. The response is preceded by an upward on-transient and followed by a downward off-transient. There is no response to light in the *ninaE^17^* mutant strain despite the use of 1000-fold brighter stimulus. As was the case with steady state protein expression levels and rhabdomere morphology, flies expressing the mutant pigments D124N, D147N, and D203N demonstrate a fully wild-type response. The response for D96N is reduced, whereas the response of E294Q is dramatically reduced (shown at 100-fold brighter stimulus).

**FIGURE 6.**
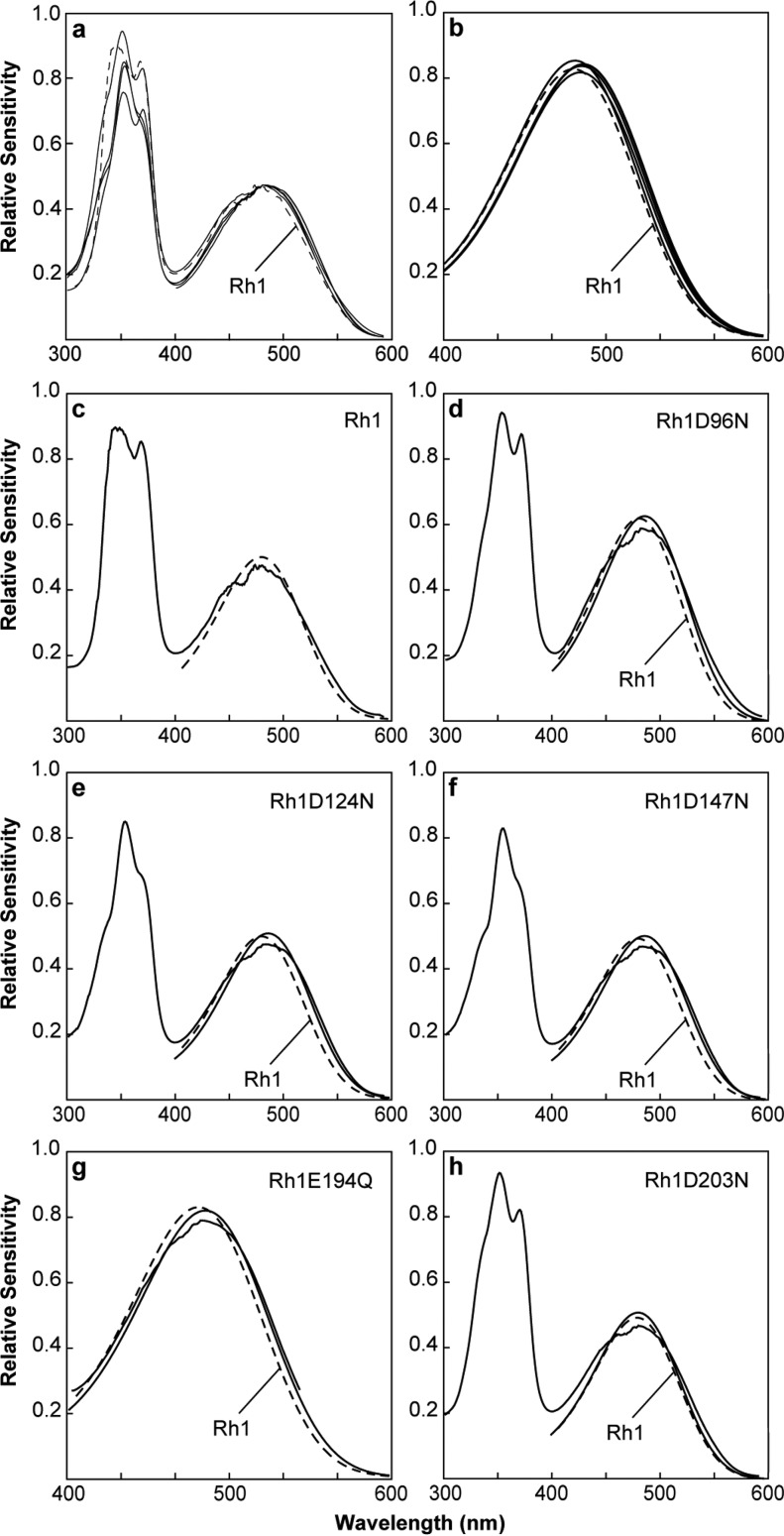
**Spectral sensitivities of flies expressing wild-type and mutant forms of Rh1.** *a–h*, measured spectral sensitivities of flies expressing Rh1, or the mutant Rh1 pigments expressed in the R1–6 photoreceptor cells. Each sensitivity spectrum obtained in this study was also fit to a rhodopsin absorption nomogram. *a,* mean spectral sensitivities of flies expressing Rh1 D96N, Rh1 D124N, Rh1 D147N, Rh1 E194Q, and Rh1 D203N (*black lines*) compared with flies expressing the unmodified Rh1 pigment (*dashed line*). All of the mutant pigments are slightly red-shifted with respect to wild-type Rh1. For all sensitivity data, the λ_max_, correlation coefficient, and number of flies examined are indicated in Table 1. The large peak in the UV region occurs because of the action of a sensitizing pigment that absorbs in the UV and activates the Rh1 rhodopsin through energy transfer. The peaks in the visible region are because of direct absorption by the visual pigment itself. *b*, the nomogram curve fits for Rh1 and the site-directed mutants demonstrate the same small red shift found in *panel a. c,* spectral sensitivity of wild-type flies expressing Rh1 in the R1–6 cells (*solid line*) with the nomogram curve fit of the data in the visible region (*dashed line*). *d–h*, spectral sensitivity measurements from animals expressing the indicated mutant form of Rh1, compared with the nomogram curve fit for the mutant pigment (*solid line*) and the nomogram curve fit for wild-type Rh1 (*dashed line*). The nomogram curve fits for Rh1 D96N, Rh1 D124N, Rh1 D147N, Rh1 E194Q, and Rh1 D203N λ_max_ are red-shifted from wild-type Rh1 at 5, 6, 6, 4, and 1 nm, respectively. The fine structure noted in the sensitivity spectra in the region of 475 nm is an artifact and results from spectral spikes in xenon lamp output in this region.

To determine the effect that these amino acid substitutions have on the absorption properties of the Rh1 visual pigment, we measured the spectral sensitivity of the transgenic animals expressing the Rh1 mutants. Fig. 6*a* shows that only a modest shift in spectral sensitivity is observed in animals expressing the mutant pigments compared with animals expressing the unmodified Rh1 pigment. The spectral sensitivity of *Drosophila* consists of two principal components. The large peak in the UV region occurs because of the action of a sensitizing pigment that absorbs in the UV and activates the Rh1 rhodopsin through energy transfer (50). The broader peak in the blue region (maximal absorption (λ_max_) = 480 nm) corresponds to the absorption of Rh1 and the direct activation of the visual pigment through absorption of a photon of light and the isomerization of the chromophore from the 11-*cis* to all-*trans* conformation. Table 1 shows λ_max_ of each mutant pigment compared with wild-type Rh1, determined from curve fitting as described under “Experimental Procedures.” Fig. 6*b* shows the underlying theoretical rhodopsin absorption curves of each of the mutants compared with wild-type Rh1 (Rh1 D96N, Rh1 D124N, Rh1 D147N, Rh1 E194Q, and Rh1 D203N). Fig. 6, *c–h*, show an individual comparison between the measured sensitivity of each mutant, its calculated absorption profile, and the calculated absorption profile of wild-type Rh1 (*dashed line* in each case). Each of the mutant pigments displays a shift in sensitivity to longer wavelengths of 1–6 nm. In the case of Rh1 E194Q, which is a mutation in the amphioxus counterion at bovine rhodopsin position Glu-181, the sensitivity of the mutant pigment is shifted to longer wavelengths (red shifted) by 4 nm (Fig. 6*g*). This contrasts significantly with the effect of the E181Q mutation in amphioxus, which resulted in a dramatic blue shift in the absorption of the pigment, of ∼100 nm. In the case of the amphioxus pigment, this large shift demonstrated that Glu-181 functions as the counterion, which we do not observe in *Drosophila*. Similarly, there is no dramatic blue shift in the sensitivity of any of the other mutants introduced at Rh1 residues Asp-96, Asp-124, Asp-147, and Asp-203 (Fig. 6, *d–f*, and *h*), respectively.

**TABLE 1:** **λ_max_ and rhodopsin absorption nomogram curve data for each wild type and modified visual pigment examined in the study**

Visual pigment	λ_max_		Correlation coefficient*^a^*		Number of flies analyzed		Figure panels
	R	M	SS	MSP	SS	MSP	
**Rh1**	480	560	0.983	0.997	3	7	Fig. 6 all Fig. 7, *a–c*
**Rh1 D96N**	485	NA*^b^*	0.991	NA	12	NA	Fig. 6, *a*, *b*, *d*
**Rh1 D124N**	486	554	0.987	0.995	8	12	Fig. 6, *a*, *b*, *e* and Fig. 7, *a*, *b*, *d*
**Rh1 D147N**	486	551	0.988	0.994	13	13	Fig. 6, *a*, *b*, *f* and Fig. 7, *a*, *b*, *e*
**Rh1 E194Q**	484	NA	0.990	NA	8	NA	Fig. 6, *a*, *b*, *g*
**Rh1 D203N**	481	555	0.977	0.993	11	10	Fig. 6, *a*, *b*, *h* and Fig. 7, *a*, *b*, *f*

*^a^* Correlation coefficient = coefficient of the fit of the nomogram curve to the absorption or sensitivity data; R = rhodopsin; M = metarhodopsin; SS = spectral sensitivity data; MSP = microspectrophotometry data.

*^b^* NA, not available (see “Results”).

**FIGURE 7.**
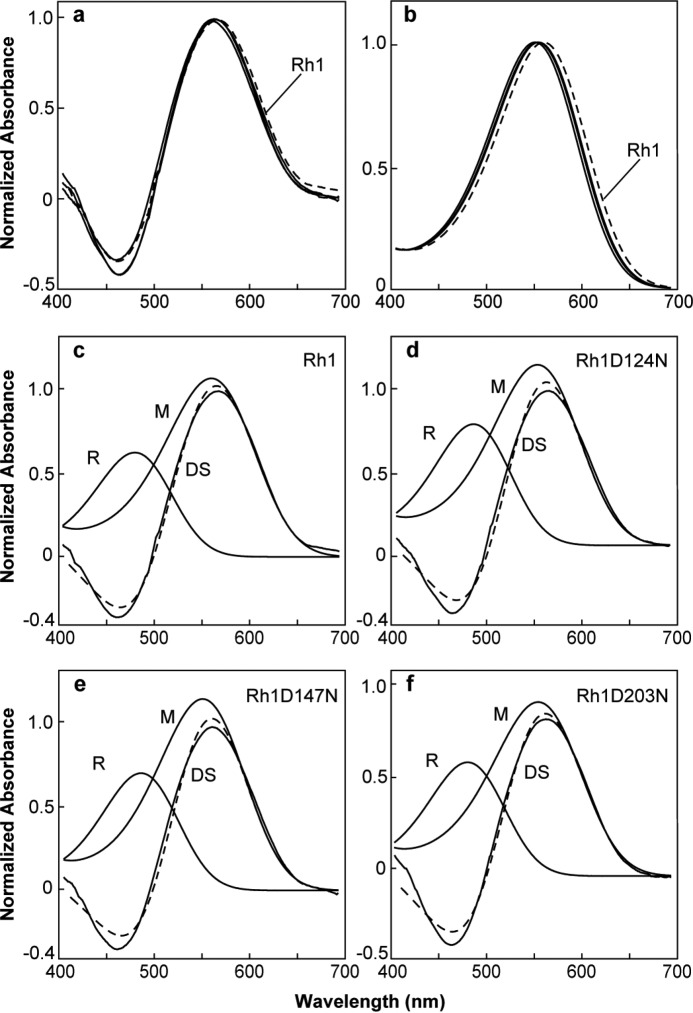
**Rhodopsin/metarhodopsin difference spectra of flies expressing wild-type and mutant forms of Rh1.** *a,* difference spectra were measured by *in vivo* MSP of flies expressing wild-type Rh1 (*dashed line*) or Rh1 D124N, Rh1 D147N, and Rh1 D203N (*solid lines*). *b-f*, calculated rhodopsin (*R*) and metarhodopsin (*M*) absorption spectra based on nomogram curve fitting to the measured difference spectra (*DS*). In all calculations, the λ_max_ for the R spectra was set to the λ_max_ measured physiologically. For MSP data, the λ_max_, correlation coefficient, and number of flies examined are indicated in Table 1. *b*, calculated M absorption spectra of wild-type Rh1 (*dashed line*) and all of the mutant pigments (*solid lines*). *c,* Rh1 measured DS (*solid line*) with calculated R and M form absorption spectra (*solid lines*) and the calculated DS (*dashed line*). As in *c*, for (*d*) Rh1 D124N, (*e*) Rh1 D147N, and (*f*) Rh1 D203N.

Photoactivation of rhodopsin occurs upon absorption of a photon by the retinal chromophore. This induces the isomerization of the 11–12-*cis* double bond to the trans configuration, which then induces a series of conformational changes in the protein that lead to its activation and the formation of metarhodopsin. *Drosophila* Rh1 is a member of the group of bistable visual pigments that do not bleach, but rather form thermally stable metarhodopsin upon illumination with blue light. Furthermore, metarhodopsin can be photoconverted back to rhodopsin by illumination with orange light, thus establishing a convenient means to measure the absorption of both rhodopsin and metarhodopsin following photoconversion *in vivo* (51). To determine the absorption profile of the metarhodopsin (M) forms of the modified Rh1 pigments, we used MSP to examine dissected retina from transgenic and wild-type flies (Fig. 7*a*). The difference spectra measured by MSP reflects the mathematical subtraction of the absorption of the visual pigment in the native state, rhodopsin form (R-form), from the absorption of the activated metarhodopsin form (M-form) of the pigment. The calculated M-form absorption of Rh1, Rh1 D124N, Rh1 D147N, and Rh1 D203N are shown in Fig. 7*b*. The absorption profiles of the mutant pigments are blue-shifted to shorter wavelengths from 5 to 9 nm. The underlying R-form and M-form absorptions of each pigment are shown in Fig. 7, *c–f*, with the calculated difference spectrum shown as a *dashed line*. We were unable to record a difference spectrum from transgenic animals expressing Rh1 E194Q and obtained only a single recording of a difference spectrum from an animal expressing Rh1 D96N, which had a calculated M-form absorption of 559 nm (data not shown). These results indicate that although amino acid substitutions at Asp-124, Asp-147, and Asp-203 are capable of altering the absorption of the M-form of the pigment, these absorption shifts are quite small.

## Discussion

The principal result from this study is that substitution of Asp or Glu amino acids present at positions 96, 124, 147, 194, and 203 in the *Drosophila* Rh1 visual pigment does not cause the dramatic spectral shifts that would be expected if one of these residues was the counterion. Substitutions at these sites with Asn or Gln cause small red shifts in the absorption of the native *R*-form of Rh1 of 1–6 nm. Our observation of a 5-nm red shift in the absorption of Rh1 D96N differs somewhat from previous studies on vertebrate visual pigments that demonstrated a 0–9-nm blue shift in D83N mutant forms of bovine rhodopsin (4, 6, 52–59). Our result of a 4-nm red shift in the absorption of Rh1 E194Q is identical to the 4-nm red shift of the E181Q mutant of bovine rhodopsin reported previously (6). The small 4-nm red shift we observed in the Rh1 E194Q mutant is in marked contrast to the effect of the E181Q mutation in amphioxus, which resulted in a dramatic blue shift in the absorption of the pigment, of ∼100 nm (10). Our result is also in stark contrast to similar studies of squid retinochrome and amphioxus peropsin, which have also been shown to have counterions present at Glu-181 (9, 10).

As we described in the Introduction, the basis for this discrepancy may be that there are substantial phylogenetic, structural, and functional differences between the G_o_-opsin of the ciliary photoreceptor cells of amphioxus and the G_q_-opsins of the rhabdomeric photoreceptors of *Drosophila*, see Fig. 2 (13–16, 19, 60). Furthermore, the squid retinochrome and amphioxus peropsin photoisomerases are substantially diverged from all of the visual pigments of vertebrates and invertebrates (60).

This raises the obvious question that if the counterion of *Drosophila* rhodopsin does not reside at the position of Glu-181 in bovine rhodopsin, and the tyrosine/phenylalanine substitution at the position corresponding to Glu-113 is not responsible for a substantial spectral shift (8), then what residue(s) or mechanisms provide this counterion function? Although modeling studies of Glu-181 in squid rhodopsin have suggested that this residue is the counterion for dark-adapted squid rhodopsin (12), previous analysis of the crystal structure suggested that Glu-181 (bovine numbering) was too distant from the Schiff base nitrogen to have a direct effect (33). In addition, these authors suggested that the hydrogen-bonding partner of the Schiff base in the dark state could be the hydroxyl group of Tyr-111 (squid numbering) or the side chain carbonyl of Asn-87 (33). These residues are conserved among invertebrate rhodopsins and correspond to Glu-113 and Gly-89 (or Gly-90 depending on the alignment) in bovine rhodopsin. In previous studies we have demonstrated that the hydroxyl group of this Tyr residue is not required in *Drosophila* Rh1 for visible sensitivity (Rh1 Y126F) (8). At the position of squid rhodopsin Asn-87, the *Drosophila* rhodopsins contain Thr, Ser, Lys, Lys, Asn, and Thr in Rh1–6, respectively. We have also shown that the Lys residue contained in Rh3 and Rh4 at this position is responsible for UV sensitivity in these pigments and that replacement of Lys with Asn or Glu is sufficient to convert Rh3 from UV to visible sensitivity (8). Perhaps the presence of Thr, Ser, Asn, or Thr play a role in stabilizing the protonated Schiff base in Rh1, -2, -5, and -6. Nonetheless, the distance of the Thr-102 hydroxyl from the Schiff base nitrogen in our *Drosophila* Rh1 model (Fig. 1, ∼13 Å), suggests that it is unlikely to have a direct effect on the chromophore.

An alternative possibility is the formation of a more complex hydrogen bonding network involving internal waters within the chromophore binding pocket in the interior of the protein. Analysis of the squid rhodopsin crystal structure demonstrated nine interhelical water molecules forming an extensive hydrogen bonding network from the chromophore binding pocket to the cytoplasmic surface (33). The authors suggest that conformational changes in this network could be important during photoactivation, although since the network begins with a series of peptide backbone carbonyls beginning with the Lys attachment site of the chromophore, this could provide a means to distribute the negative charge of the protonated Schiff base as well. The involvement of internal water is particularly attractive in such a model, due to molecular dynamics studies demonstrating spectral shifts of up to 34 nm due to a single water molecule, depending on the environment of the chromophore (61). A further possibility is that there is a binding site for chloride or another anion in the protein that serves as the counterion. Work on the vertebrate visual pigments has implicated amino acids at residues 181, 289, and 292 as being involved in this process (62, 63). As noted earlier, *Drosophila* Rh1 contains a Glu rather than His at position 181 (bovine numbering), so this site is unlikely. We have observed a shift of 10–17 nm caused by a Ser/Ala substitution at the position of Ala-292 (28), although this seems unlikely to explain a potential counterion effect. Another possible explanation for our results is potential compensation in the E194Q mutant by physiological Cl^−^. Addition of 100 mm NaCl to the bovine rhodopsin counterion mutant E113Q was observed to reverse the effect of the mutation, shifting absorption of the pigment from 380 to 495 nm (6). The concentration of Cl^−^ in adult *Drosophila* hemolymph is ∼60 mm (64), and could potentially obscure a counterion effect. Finally, the mutant E194Q pigment could adopt an alternate configuration, which allows recruitment of an alternate counterion. We have observed spectral evidence suggesting the simultaneous adoption of two spectral (conformational) states in a mutant of Rh3 (F133E) (8).

Although we have demonstrated that the E194Q mutant (at the site of bovine Glu-181) does not cause a dramatic spectral shift in the ground state of the pigment, previous studies of bovine rhodopsin have demonstrated that this residue functions as a counterion for the protonated Schiff base of the activated photoproduct (11). Indeed, modeling studies have suggested that the corresponding residue in the mouse UV cone pigment is likely to function as the counterion of the activated photoproduct metarhodopsin I (65). Interestingly, the E181Q mutant displays enhanced reactivity to hydroxylamine, and has an increased Meta II decay rate (66). In addition, the E181Q mutant has also been shown to undergo accelerated decay of bathorhodopsin and in the presence of chloride ion shows an increased lumirhodopsin I-lumirhodopsin II spectral shift and delayed deprotonation of the Schiff base (67). These findings are consistent with the idea that Glu-181 plays an important role in the early stages of rhodopsin activation and that a negative charge at this position stabilizes the protonated Schiff base in later photoactivated intermediates.

Consistent with the importance of Glu-181 in rhodopsin function is the observation that human mutations in rhodopsin at this site (E181K) are associated with autosomal dominant retinitis pigmentosa and have been identified repeatedly in diverse populations (68–70). When expressed *in vitro* the E181K mutant pigment is unable to bind chromophore (66). Indeed the corresponding mutant in *Drosophila* Rh1 E194K was recovered in a screen for dominant retinal degeneration mutants (71). In characterizing the *Drosophila* Rh1 E194Q mutant we have found that newly eclosed adults expressingthe mutant pigment have a measureable light response, but that this response decreases with time so that mature animals have dramatically reduced light response 7 days after eclosion (Fig. 5). Furthermore, animals expressing the E194Q mutant also undergo dramatic degeneration of rhabdomere morphology in the R1–6 photoreceptor cells (Fig. 4). These findings and our inability to determine the absorption of metarhodopsin by MSP may reflect 1) a requirement for Glu-181 to form a normal functional visual pigment or, 2) an intrinsic instability of the activated pigment in the Rh1 E181Q mutant because of a defect in the counterion of metarhodopsin.

The experiments in the present study represent the first direct test of the hypothesis that Glu-181 functions as the counterion in a G_q_-coupled invertebrate rhodopsin. Our finding that the *Drosophila* E194Q mutant does not show a dramatic blue shift in the absorption of the dark adapted pigment is consistent with the idea that this residue is not required as a counterion in this pigment, although we discussed a series of alternative interpretations. Similar experiments performed on mouse melanopsin have also demonstrated that the residue corresponding to Glu-181 is not required as a counterion in melanopsin, discussed in Ref. 72. Melanopsin is a non-image forming rhodopsin expressed in retinal ganglion cells and has been shown to regulate circadian photoentrainment and pupil constriction (73, 74). Phylogenetically, melanopsin is more closely related to the invertebrate than the vertebrate visual pigments, and is thought to activate a phospholipase C-mediated signaling cascade through a G_q_ G-protein (Fig. 2) (19, 73). In conclusion, our results demonstrate that substitution of Glu-181 with glutamine does not produce a large blue shift in the absorption of the pigment, as would be expected for a potential counterion. Definitive identification of the counterion in *Drosophila* rhodopsin will require further study.

## Author Contributions

S. G. B. conceived and coordinated the study, performed the electrophysiology experiments shown in Fig. 5, and wrote the paper. L. Z. performed the electrophysiology and microspectrophotometry experiments shown in Figs. 6 and 7. D. M. F. performed the molecular modeling upon which Fig. 1 is based. R. M. F., E. E. B., and E. S. built the mutant Rh1 transgenes, obtained the transgenic lines, and performed genetic procedures. M. M. performed the experiments shown in Figs. 3 and 4 and performed genetic procedures. All authors reviewed the results and approved the final version of the manuscript.

## Footnotes

2 The abbreviations used are:

Rh1: rhodopsin 1
MSP: microspectrophotometry.
